# First-time incidence of ischemic heart disease among immigrants in Sweden: variation by country of origin, duration of residence, and immigrant generation in a nationwide cohort study

**DOI:** 10.1093/eurpub/ckag192

**Published:** 2026-11-06

**Authors:** Daniel Nigusse Tollosa, Kazem Zendehdel, Sol P Juarez, Alexander Miething, Andrea Dunlavy, Mikael Rostila

**Affiliations:** Department of Public Health Sciences, Stockholm University, Stockholm, Sweden; Cancer Research Center, Cancer Institute, Tehran University of Medical Sciences, Tehran, Iran; Department of Medical and Surgical Sciences, University of Bologna, Bologna, Italy; Department of Public Health Sciences, Stockholm University, Stockholm, Sweden; Centre for Health Equity Studies, Stockholm University/Karolinska Institutet, Stockholm, Sweden; Department of Public Health Sciences, Stockholm University, Stockholm, Sweden; Centre for Health Equity Studies, Stockholm University/Karolinska Institutet, Stockholm, Sweden; Department of Public Health Sciences, Stockholm University, Stockholm, Sweden; Centre for Health Equity Studies, Stockholm University/Karolinska Institutet, Stockholm, Sweden; Department of Public Health Sciences, Stockholm University, Stockholm, Sweden; Centre for Health Equity Studies, Stockholm University/Karolinska Institutet, Stockholm, Sweden; Aging Research Center, Karolinska Institutet/Stockholm University, Stockholm, Sweden

## Abstract

As Sweden’s immigrant population has become increasingly diverse and many countries of origin have undergone an epidemiological transition with rising cardiovascular disease (CVD) risk factors, understanding disparities in ischemic heart disease (IHD) among immigrants and their descendants has become increasingly important. This nationwide registry-based cohort study included over 8.5 million individuals aged 35–85 years [50.1% women; 18.2% first-generation (G1) and 9.4% second-generation (G2) immigrants] who followed from 1993 to mid-2023. Adjusted Cox regression models were used to examine associations between migrant-related factors—including country of origin, duration of residence, period of immigration, and immigrant generation—and first-time IHD incidence. During follow-up, 703 212 first-time IHD events occurred. Compared with Swedish-born individuals, G1 immigrants from the Nordic countries, the Middle East, and selected Asian countries (Afghanistan, Pakistan, India, and Sri Lanka), as well as G2 individuals with Finnish parental origin, had a higher risk of IHD. The highest risks were observed among recent immigrants from the Middle East and among immigrants with the shortest duration of residence. Risk generally declined with increasing duration of residence and became lower than that of the reference population in many immigrant groups. Although age-standardized IHD incidence rates declined over the past three decades, the pace of decline varied across immigrant groups and was slowest among Asian immigrants. In conclusion, most immigrant groups, particularly recent immigrants from the Middle East and immigrants with the shortest duration of residence, had a higher risk of IHD. Tailored cardiovascular prevention strategies are essential to reduce these disparities among immigrants in Sweden.

## Introduction

Cardiovascular disease (CVD) remains the leading cause of mortality worldwide, accounting for approximately 20 million deaths in 2023 [1]. Among CVDs, ischemic heart disease (IHD) is the largest contributor to the global CVD burden and the leading cause of premature cardiovascular mortality [2, 3]. Although substantial reductions in IHD incidence and mortality have been achieved in many high-income countries over recent decades, including Sweden, IHD continues to impose a considerable public health burden and contributes substantially to socioeconomic inequality in health [3–5]. As populations become increasingly diverse through international migration, understanding inequalities in IHD has become an important public health priority.

Migrants often experience different risks of IHD than host populations because they are exposed to distinct socioeconomic, environmental, behavioral, and healthcare contexts before, during, and after migration [6–8]. A systematic review of migrant populations in Europe, North America, and other high-income countries found that several immigrant groups have exhibited a similar or higher risk of IHD than native-born populations, although considerable heterogeneity exists across receiving countries and migrant groups [6, 7, 9]. This heterogeneity likely reflects variation in migrants’ countries of origin and in the social, healthcare, and epidemiological context of the receiving countries [8, 10].

In recent decades, many low- and middle-income countries have undergone epidemiological transitions, with rapid increases in CVD risk factors, including obesity, diabetes, hypertension, and dyslipidaemia [11–15]. Consequently, migrants from some regions, such as the Middle East, Central and South Asia, and parts of Eastern Europe, may arrive with a higher baseline cardiovascular risk profile. Following migration, acculturation-related lifestyle changes and psychosocial stress associated with the integration process may further modify CVD health [10]. However, the magnitude and direction of these changes are likely determined by both individual characteristics and structural factors, including migration policies, access to healthcare, and employment opportunities. The intergenerational transmission of CVD risk has also been documented, suggesting that the cardiovascular health of second-generation immigrants may be influenced by parental cardiovascular risk factors and health behaviors [16, 17]. Conversely, the healthy-migrant effect proposes that migrants are initially healthier than the host population because healthier individuals are more likely to migrate, with health gradually converging toward that of the host population over time [18]. However, evidence supporting this hypothesis is inconsistent and appears to vary according to country of origin, migration pathway, and duration of residence (DoR).

Sweden provides an important setting in which to investigate these questions. Nearly one-third of the Swedish population consists of first- or second-generation immigrants [19], with considerable diversity in geographic origin, migration history, culture, and socioeconomic circumstances. Swedish immigration patterns have also changed considerably over time, shifting from predominantly labor-driven migration during the 1960s and 1970s, which primarily involved young and healthy immigrants, to increasing humanitarian and family-related migration, attracting individuals from diverse regions with different CV risk profiles. These changes in migration patterns, together with the epidemiological transition occurring in many countries of origin, underscore the need for updated evidence on IHD among immigrants in Sweden. Moreover, previous studies have largely focused on broad immigrant populations or selected regions of origin, with limited attention to how IHD risk varies simultaneously by country of origin, DoR, and immigrant generation within a single nationwide population. Therefore, this study aimed to examine the incidence of first-time IHD among first- and second-generation immigrants, examining differences according to country of origin, DoR, immigration cohort, and intergenerational differences.

## Methods

### Study population

This study was based on data from multiple Swedish national registers linked using pseudonymized personal identity numbers. Information on participants’ and their parents’ country of birth, together with socioeconomic and demographic characteristics, was obtained from the Total Population Register [20], the Longitudinal Integrated Database of Health Insurance, and Labor Market Studies (LISA) [21], and the Multi-Generation Register [22]. Information on first-time IHD, including the date of diagnosis and corresponding ICD codes, was obtained from the National Patient Register [23], while mortality data were retrieved from the Cause of Death Register [24].

Given that IHD is most commonly diagnosed in mid-adulthood and many cardiovascular risk factors become clinically apparent during this period, age 35 years was chosen as the start of follow-up. An open cohort design was applied, with follow-up beginning on 1 January 1993 for individuals aged ≥35 years who were residing in Sweden at baseline. Individuals who reached age 35 during the study period entered the cohort at that age. First-generation immigrants who arrived in Sweden at age ≥35 years were eligible for follow-up after a 5-year buffer period. Thus, individuals who arrived on or after 1 January 1988 entered follow-up 5 years after immigration, whereas those who arrived before this date entered follow-up at the study baseline (1 January 1993). This buffer period was applied to reduce the likelihood of including prevalent IHD cases diagnosed shortly after immigration and to allow sufficient time for the identification of first-time IHD within the Swedish healthcare system.

Follow-up continued until the first occurrence of IHD, emigration, death, reaching 85 years of age, or the end of follow-up (mid-2023), whichever occurred first. Follow-up was restricted to age 85 because of the limited number of events among older immigrants and to focus on the age groups most relevant for primary CVD prevention. Figure 1 illustrates the flowchart of the study population selection.

**Figure 1:**
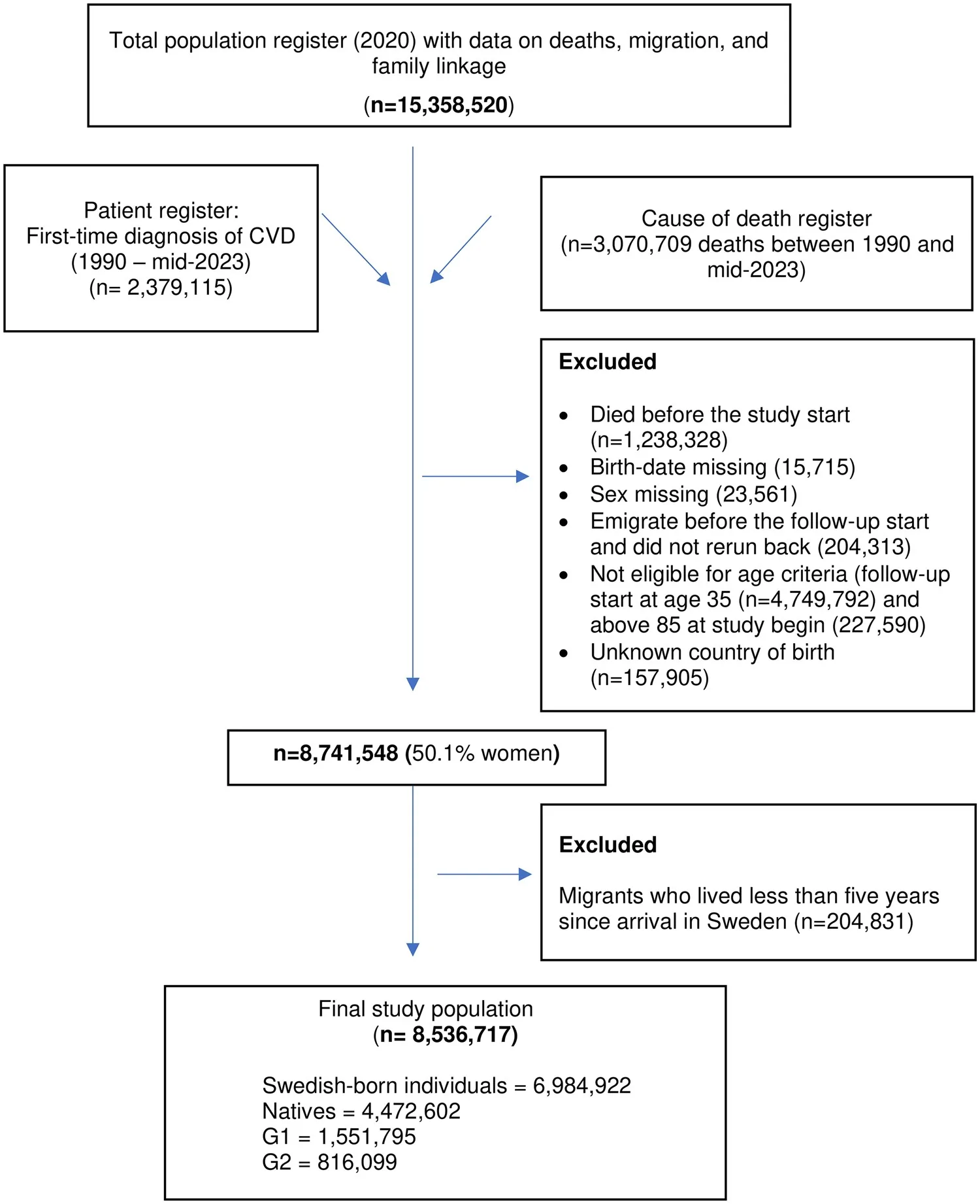
Flowchart illustrates the inclusion and exclusion criteria for study population.

### Assessment of exposure and outcome variables

As exposure variables, we identified first-generation immigrants (born outside Sweden; G1) and second-generation immigrants (born in Sweden with at least one foreign-born parent; G2). Individuals were classified according to their own region of origin for G1 or their parent’s region of origin for G2, using the following categories: (i) Nordic countries excluding Sweden; (ii) the European Union (EU) and the United Kingdom (UK), excluding the Nordic countries; (iii) other European excluding the Nordic countries, the EU and the UK; (iv) the Middle East; (v) Africa; (vi) Asia; (vii) North America; and (viii) Latin America. Country-specific estimates within these regions were also reported for countries with sufficiently large numbers of G1 and G2 individuals and/or a substantial burden of IHD.

First-generation immigrants were further categorized by year of arrival in Sweden (before 1970, 1971–2000, 2001–10, and 2011–18) and by DoR, calculated as the interval between the date of arrival and the end of follow-up (5–15, 16–30, ≥31, in years). These categories were selected to reflect major phases in Sweden’s immigration history, including earlier labor migration and later increases in humanitarian and family reunifications, and therefore broadly correspond to distinct migration contexts.

Covariates included individual disposable income (categorized into quintiles), highest educational attainment (primary, secondary, tertiary, and missing/unknown), occupation class (using the International Standard Classification of Occupation [ISCO-08] and grouped into four categories: high-skilled white-collar, low-skilled white-collar, high-skilled blue-collar, and low-skilled blue-collar), marital status (not-married/single, married, divorced, or widowed), and healthcare regions based on residential county (Stockholm-Götland, Uppsala-Örebro, Southeastern, Southern, Western, and Northern regions), reflecting the organization of the Swedish healthcare system and accounting for potential regional differences in healthcare access, quality, and resource distribution. Detailed definitions of all covariates and their analytical categories are provided in Table S1.

The outcome was the first recorded diagnosis of IHD, identified from the Swedish National Patient Register using ICD-9 codes 410–414 (1993-1996) and ICD-10 codes I20–I25 (1997 onwards), including both acute and chronic IHDs.

### Statistical analysis

Sex-stratified Cox proportional hazards regression models, using age as the underlying time scale, were fitted to estimate the association between immigrant status and incidence IHD. Results are presented as hazard ratios (HRs) with 95% confidence intervals (CIs). Two models were fitted. Model 1 adjusted for calendar period, which was treated as a time-varying covariate by splitting each participant’s follow-up into 5-year calendar intervals (1993–97, 1998–2002, 2003–07, 2008–12, 2013–17, and 2018–mid-2023). Model 2 additionally adjusted for socioeconomic and demographic factors, including individual disposable income, education attainment, occupational class, marital status, and healthcare region. We also evaluated alternative operationalizations for income, educational attainment, and occupational class, using their values at cohort entry and the highest levels recorded during follow-up. These alternative definitions were considered to better capture long-term socioeconomic position rather than temporary socioeconomic disadvantage, particularly among recently arrived immigrants. Model fit was compared using the Akaike information criterion and Bayesian information criterion, and the operationalization with the better fit was retained in the model. Marital status and healthcare region were based on information recorded at cohort entry. Swedish-born individuals, including second-generation immigrants (G2), were used as the reference group for comparisons with the first-generation immigrants (G1), who were further stratified by country of origin and DoR. Native Swedes (individuals born in Sweden to two Swedish-born parents) were used as the reference group for comparisons with the second-generation immigrants (G2).

Age-standardized incidence rates (ASIRs) were calculated per 100 000 person-years using the direct standardization method, with the 2013 European Standard Population as the reference population. ASIRs were estimated separately for each 5-year calendar period (1993–97, 1998–2002, 2003–07, 2008–12, 2013–17, and 2018─mid-2023) to describe the temporal trend in IHD incidence. To summarize the overall trend across the entire study period, the average annual percentage change (AAPC) was estimated. The AAPC was calculated as a weighted average of the annual percentage changes, with weights proportional to the length of each time interval, providing a single summary measure of the overall temporal trend in ASIRs.

## Results

### Participant characteristics

The study included 8 536 717 individuals, of whom 50.1% were women, 18.2% were G1, 9.4% were G2, and 52.4% were native Swedes. During follow-up (1993–2023), 703 212 incidence IHD cases were identified, of which 12.9% occurred among G1, 8.5% among G2, and 37.7% among native Swedes. Overall, 64.5% of IHD incidence cases occurred in men. At cohort entry, G1 immigrants were younger than Swedish-born individuals (mean age 42.6 [SD 11.0] vs. 47.6 [SD 14.0]). Socioeconomic profiles between G2 and native Swedes were somewhat comparable, whereas, compared with Swedish-born individuals, G1 had lower disposable income, a higher proportion of married individuals, and was more likely to be employed in low-skilled occupations (Table S2).

### IHR by country/region and generation group

Table 1 presents the association between first-time IHD incidence and immigrant generation—further stratified by individuals’ and/or parental country of origin. In the fully adjusted model, first-generation (G1) immigrants had a higher risk of IHD compared with Swedish-born individuals—with HRs of 1.16 (95% CI: 1.14–1.17) in women and 1.14 (95% CI: 1.13–1.15) in men, indicating that these differences were not explained by socioeconomic and demographic factors. The corresponding ASIRs per 100 000 person-years were 363.8 and 753.2 among G1 women and men, respectively, compared with 314.1 and 689.9 among Swedish-born women and men, respectively.

**Table 1:** Number of cases, ASIR per 100 000 person-years, and hazard ratios (HRs) with 95% confidence interval (CIs) for first-time IHR among individuals aged 35–85 years in Sweden, by immigrant generation, country or region of origin, and sex, 1993 to mid-2023.

Countries or regions	Women			Men		
	Cases (in 10^5^ person-years)	ASIR	HRs (95% CIs)	Cases (in 10^5^ person-years)	ASIR	HRs (95% CIs)
Swedish-born	216 684 (630.8)	314.1	Ref	395 774 (604.4)	689.9	Ref
All G1	33 329 (107.6)	363.8	1.16 (1.14–1.17)	57 435 (95.3)	753.2	1.14 (1.13–1.15)
Nordic excl. Sweden	17 851 (34.9)	436.4	1.29 (1.27–1.31)	22 606 (25.5)	853.5	1.18 (1.17–1.20)
Finland	13 275 (25.7)	448.9	1.36 (1.33–1.38)	15 857 (17.4)	885.7	1.25 (1.23–1.27)
Denmark	1763 (3.9)	365.1	1.09 (1.04–1.14)	3 597 (4.3)	751.7	1.02 (0.98–1.05)
Norway	2757 (4.9)	427.4	1.14 (1.09–1.18)	3018 (3.4)	802.2	1.13 (1.09–1.17)
EU and UK excl. Nordic	6969 (21.6)	320.8	1.03 (0.99–1.07)	12 360 (19.1)	696.6	1.03 (1.01–1.05)
Poland	1947 (6.8)	373.3	1.25 (1.19–1.31)	2272 (3.9)	854.5	1.33 (1.28–1.39)
Hungary	650 (1.6)	374.0	1.17 (1.09–1.27)	1442 (1.6)	801.7	1.09 (1.03–1.14)
Romania	317 (1.6)	284.4	0.93 (0.83–1.04)	592 (1.3)	626	0.98 (0.91–1.05)
Other European (non-EU)	3088 (10.9)	352.1	1.10 (1.06–1.14)	6219 (9.4)	771.6	1.14 (1.11–1.17)
Russia	95 (1.1)	225.7	0.76 (0.63–0.94)	101 (0.4)	588.6	1.13 (0.93–1.37)
Former Jugoslavia	2013 (6.5)	361.8	1.10 (1.06–1.15)	4335 (6.5)	744.5	1.12 (1.08–1.16)
Former Czechoslavkia	323 (0.9)	309.8	1.06 (0.95–1.18)	667 (0.7)	781.5	1.13 (1.04–1.21)
Former Soviet union	477 (0.9)	433.5	1.31 (1.20–1.43)	515 (0.5)	928.2	1.18 (1.08–1.28)
Middle East	3389 (17.3)	376.5	1.18 (1.14–1.22)	10 786 (21.6)	788.2	1.47 (1.44–1.50)
Turkey	844 (3.2)	426.8	1.21 (1.13–1.29)	2160 (3.7)	824.6	1.44 (1.38–1.50)
Iraq	818 (4.9)	351.0	1.17 (1.09–1.26)	2946 (6.4)	769.7	1.46 (1.41–1.51)
Iran	926 (4.8)	352.4	1.16 (1.09–1.25)	2735 (5.7)	717.1	1.37 (1.32–1.43)
Lebanone	273 (1.6)	378.9	1.17 (1.04–1.32)	1043 (2.2)	858.6	1.55 (1.46–1.65)
Syria	414 (2.3)	376.5	1.23 (1.12–1.36)	1457 (2.7)	898.1	1.74 (1.65–1.83)
Africa	349 (5.9)	148.7	0.61 (0.55–0.68)	1737 (7.6)	407.1	0.79 (0.75–0.83)
Asia	915 (10.2)	222.3	0.78 (0.73–0.83)	2200 (6.0)	644.9	1.17 (1.12–1.22)
Afghanistan	108 (0.6)	386.9	1.38 (1.15–1.67)	241 (0.6)	717.4	1.42 (1.25–1.61)
India	131 (0.9)	360.7	1.27 (1.07–1.51)	442 (0.9)	873.1	1.48 (1.35–1.62)
Pakistan	85 (0.3)	558.6	1.79 (1.45–2.22)	353 (0.6)	1219.2	1.93 (1.74–2.14)
Sri Lanka	77 (0.4)	422.7	1.26 (0.99–1.56)	132 (0.3)	816.2	1.63 (1.37–1.91)
North America	123 (1.2)	224.1	0.78 (0.66–0.94)	300 (1.3)	554.1	0.83 (0.74–0.93)
Latin America	624 (5.3)	179.0	0.72 (0.66–0.77)	1183 (4.7)	383.7	0.69 (0.65–0.73)
Natives	77 029 (451.2)	206.9	Ref	188 558 (451.4)	507.3	Ref
All G2	19 032 (79.3)	256.5	1.11 (1.09–1.14)	41 639 (77.7)	598.3	1.08 (1.06–1.09)
Nordic	3204 (26.5)	200.4	1.14 (1.09–1.20)	8202 (27.2)	503.1	1.07 (1.04–1.11)
EU and UK	949 (11.0)	162.2	1.05 (0.96–1.15)	2866 (11.5)	430.8	1.03 (0.97–1.08)
Other Europe	209 (2.4)	174.3	1.10 (0.91–1.34)	562 (2.5)	461.4	1.12 (0.99–1.26)
Middle East	20 (0.6)	572.3	1.58 (0.85–2.94)	60 (0.6)	441.8	1.31 (0.89–1.93)

HRs adjusted for calendar period (as time-varying covariate) and socioeconomic factors (disposable income, educational attainment, marital status, occupation class, and healthcare regions).

A notable difference emerged when analyzing incidence rates by region of birth. Immigrants from the Nordic countries, the Middle East, and parts of European countries had a higher risk of IHD, whereas those from Asia, Africa, North America, and Latin America generally had a lower risk. However, country-specific analyses showed substantial heterogeneity within regions. For example, despite the overall lower risk among Asian immigrants, immigrants from South Asia (Afghanistan, India, Pakistan, and Sri Lanka) exhibited significantly elevated risks, with the highest risk was observed among Pakistani immigrants (HR: female = 1.79, 95% CI: 1.45–2.22, and men = 1.93, 95% CI: 1.74–2.14). Among second-generation immigrants, no significant difference in IHD risks was observed compared with native Swedes, except among individuals with Nordic parental origin, who had a modestly elevated risk (HR: female = 1.14, 95% CI: 1.09–1.20, and male = 1.07, 95% CI: 1.04–1.11). Table S3 compares partially adjusted (Model 1) and fully adjusted (Model 2) HRs for IHD by immigrant generation, country/region of origin, and sex.

### IHR by DoR and immigration cohort

Table 2 presents the association between DoR and first-time IHD incidence among first-generation (G1) immigrants compared with Swedish-born individuals. In the fully adjusted model, G1 immigrants with the shortest DoR (5–15 years) had the highest risk of IHD (HR: female = 3.67, 95% CI: 3.53–3.82; men = 4.13, 95% CI: 4.03–4.25). This pattern was consistent across all regions of origin, with the highest risks observed among immigrants from non-EU European countries (HR: female = 3.69, 95% CI: 3.36–4.06; male = 5.37, 95% CI: 5.02–5.74) and the Middle East (HR: female = 3.73, 95% CI: 3.52–3.95; male = 4.79, 95% CI: 4.62–4.97), among those residing in Sweden for 5–15 years. Within each origin group, HRs generally declined with increasing DoR, with immigrants residing in Sweden for 31 years or more exhibiting the lowest hazards of IHD relative to the Swedish-born individuals. Table S4 presents a comparison of partially adjusted (Model 1) and fully adjusted (Model 2) HRs for IHD by DoR, country/region of origin, and sex among first-generation immigrants.

**Table 2:** Number of cases, ASIR per 100 000 person-years, and hazard ratios (HRs) with 95% confidence interval (CIs) for first-time IHR among first-generation (G1) immigrants aged 35–85 years in Sweden, by duration of residence, region of birth, and sex, with Swedish-born individuals as the reference group, 1993 to mid-2023.

Region of birth	DoR (in years)	Women			Men		
		Cases (in 10^5^ person-years)	ASIR	HRs (95% CIs)	Cases (in 10^5^ person-years)	ASIR	HRs (95% CIs)
All G1	5–15	3356 (8.9)	876.5	3.67 (3.53–3.82)	7630 (9.9)	1566.2	4.13 (4.03–4.25)
	16–30	6399 (25.1)	469.0	1.79 (1.74–1.84)	15 033 (22.6)	1078.1	2.28 (2.24–2.32)
	31+	19 483 (69.5)	292.9	0.98 (0.96–0.99)	30 275 (60.0)	573.4	0.86 (0.85–0.87)
Nordic excl. Sweden	5–15	395 (0.5)	1,064	3.46 (3.13–3.83)	845 (0.7)	1619.6	3.04 (2.83–3.26)
	16–30	1993 (2.1)	1,250	3.97 (3.79–4.15)	3780 (2.5)	1823.6	3.26 (3.15–3.37)
	31+	12 849 (30.0)	365.8	1.15 (1.13–1.17)	15 617 (21.1)	704.6	1.01 (0.99–1.02)
EU and UK excl. Nordic	5–15	605 (1.9)	689.4	2.54 (2.34–2.76)	1265 (2.4)	1141.4	2.57 (2.42–2.73)
	16–30	1224 (4.6)	463.7	1.55 (1.47–1.64)	2302 (4.0)	970.9	1.93 (1.86–2.02)
	31+	3968 (13.9)	251.3	0.86 (0.83–0.88)	7154 (11.6)	559.7	0.83 (0.81–0.85)
Other European (non-EU)	5–15	464 (0.7)	1,270.8	3.69 (3.36–4.06)	907 (0.6)	2576.9	5.37 (5.02–5.74)
	16–30	873 (4.2)	376.2	1.33 (1.24–1.43)	1860 (3.3)	889.7	1.72 (1.65–1.81)
	31+	1542 (5.9)	266.5	0.84 (0.80–0.88)	3143 (5.2)	581.6	0.81 (0.78–0.84)
Middle East	5–15	1339 (2.2)	1,208.4	3.73 (3.52–3.95)	3415 (2.9)	2147.4	4.79 (4.26–4.97)
	16–30	1469 (6.2)	428.3	1.24 (1.18–1.31)	4874 (7.1)	1115.8	2.08 (2.02–2.14)
	31+	575 (8.8)	144.0	0.47 (0.43–0.51)	2458 (11.5)	375.5	0.62 (0.60–0.65)
Africa	5–15	119 (1.3)	260.2	1.13 (0.95–1.36)	397 (1.6)	581.3	1.50 (1.36–1.66)
	16–30	140 (2.2)	152.0	0.67 (0.56–0.79)	691 (2.4)	560.8	1.41 (1.30–1.52)
	31+	78 (2.3)	105.6	0.32 (0.26–0.40)	619 (3.6)	297.3	0.46 (0.42–0.49)
Asia	5–15	258 (1.9)	568.2	1.81 (1.59–2.04)	563 (1.2)	1315.3	3.00 (2.76–3.27)
	16–30	373 (4.1)	254.8	0.81 (0.73–0.89)	881 (1.9)	854.3	1.71 (1.61–1.84)
	31+	259 (4.2)	133.5	0.47 (0.42–0.54)	706 (2.9)	428.7	0.65 (0.60–0.69)
North America	5–15	17 (0.1)	476.5	1.47 (0.92–2.37)	54 (0.2)	1408.9	2.28 (1.75–2.98)
	16–30	34 (0.4)	201.2	0.88 (0.63–1.23)	104 (0.5)	599.2	1.12 (0.92–1.36)
	31+	42 (0.6)	154.6	0.52 (0.38–0.69)	100 (0.6)	417.8	0.49 (0.41–0.60)
Latin America	5–15	153 (0.3)	993.3	2.76 (2.35–3.24)	178 (0.3)	1626	2.59 (2.24–3.01)
	16–30	293 (1.2)	347.0	1.12 (0.99–1.26)	531 (0.9)	860.5	1.50 (1.38–1.64)
	31+	165 (3.7)	77.8	0.29 (0.25–0.34)	455 (3.5)	233.5	0.36 (0.33–0.40)

HRs adjusted for calendar period and sociodemographic and socioeconomic factors (disposable income, educational attainment, marital status, occupation class, and healthcare regions).

Analysis by immigration cohort (calendar period of immigration) showed that Middle Eastern men arriving between 2011 and 2018 had the highest risk of IHD (HR = 1.89, 95% CI: 1.75–2.04), with risks increasing across successive immigration cohort. Asian males also consistently exhibited elevated risks (HRs ranging from 1.16 to 1.30 across arrival periods), whereas risks were generally lower among women, especially those arriving after 2000. The lowest risks were observed among African immigrant cohorts (Fig. 2).

**Figure 2:**
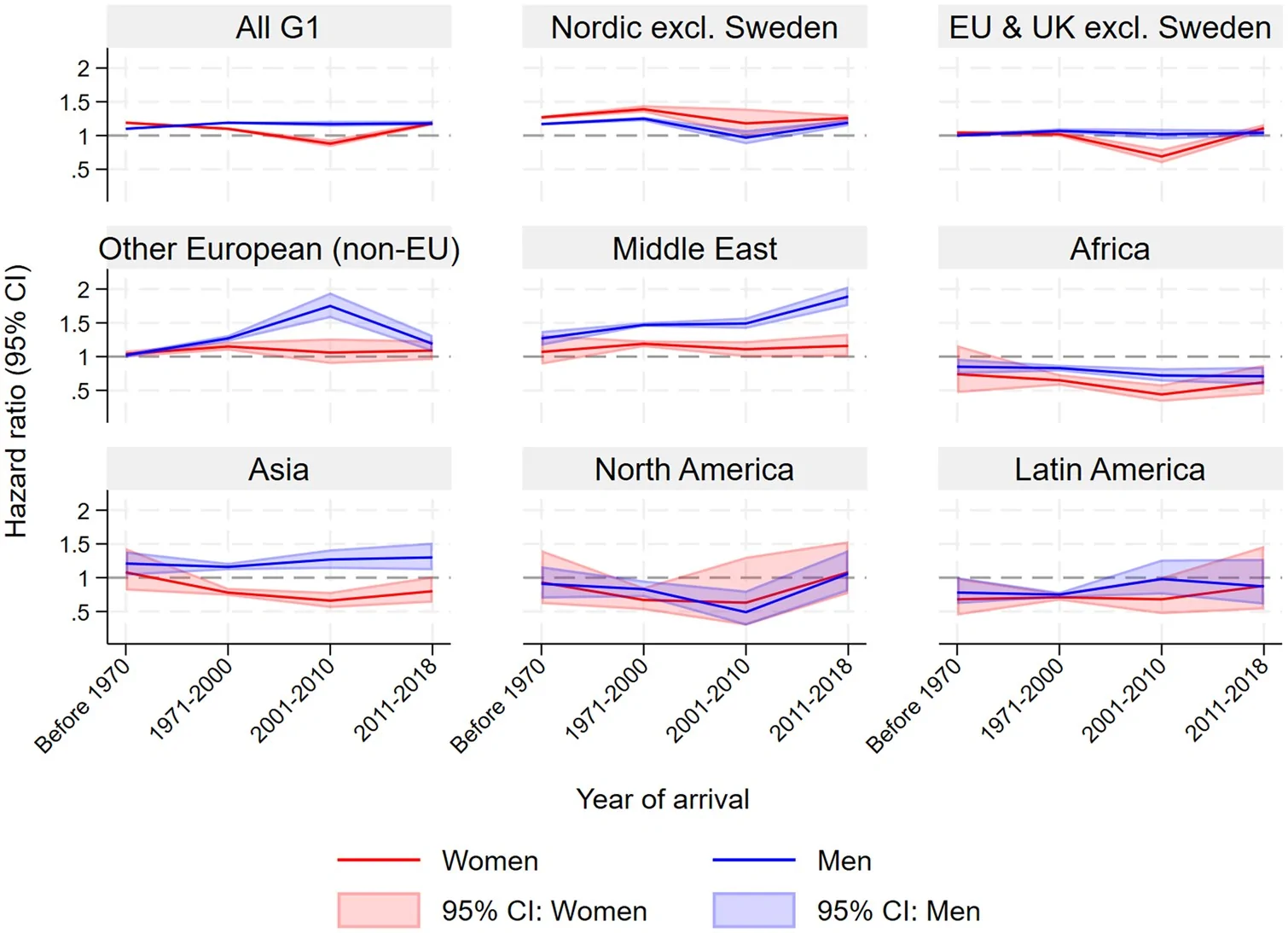
Hazard ratios for first-time IHR among first-generation immigrants aged 35–85 years in Sweden, by region of origin, year of arrival, and sex, with Swedish-born individuals as the reference group, 1993 to mid-2023.

### Temporal trend of ASIR for IHD

Temporal trends in age-standardized first-time IHD incidence showed that rates declined at somewhat similar pace among all immigrants combined (AAPC: women = −4.5% and men = −4.6%) and Swedish-born individuals (AAPC: women = −4.3% and men = −4.5%). However, the magnitude of the decline varied across immigrant groups, with the slowest reductions among Asian immigrants (AAPC: women = −2.26% and men = −3.22%). Throughout the study period, ASIRs remained highest among immigrants from the Middle East and Nordic countries (Fig. S1).

## Discussion

This study explored first-time IHD incidence among first-generation immigrants (G1; foreign-born individuals) in Sweden, considering variation by country of origin, DoR, immigration cohort, as well as among second-generation immigrants (G2; Swedish-born individuals with at least one foreign-born parent). The findings indicate that first-generation immigrants (G1) generally have a higher risk of IHD than the host population, particularly those originating from the Nordic countries, the Middle East, and South Asia. Although this excess risk tended to decline with increasing DoR, it remained elevated for decades after arrival before gradually converging with, or in some groups even becoming lower than, that of the host population. These findings are generally consistent with a systematic review of IHD risk among immigrants in Europe [6] and with previous studies from Sweden and Denmark, which reported a higher risk of coronary heart disease among immigrants [25, 26] and non-Western immigrants [27], respectively, compared with the host population. A modestly elevated risk of IHD was also observed among second-generation (G2) individuals of Nordic parental origin compared with native Swedes. Overall, our findings suggest that inequalities in IHD risk persist for many years after migration in several immigrant groups, although their magnitude varies by country of origin and immigrant generation. This indicates that different immigrant populations may benefit from tailored public health strategies, including early cardiovascular risk assessment and prevention among recently arrived first-generation immigrants, as well as continued cardiovascular health promotion in second-generation groups with persistently elevated risk.

The observed differences in IHD risk by DoR suggest that the elevated risk of IHD among first-generation immigrant groups is likely explained by a combination of premigration and postmigration factors. This pattern differs from the classical convergence hypothesis associated with the “healthy-migrant effect” phenomenon—whereby immigrants are expected to arrive with a health advantage that gradually converges toward that of the host population over time [6, 18]. One explanation is that cardiovascular risk may reflect the combined influence of premigration and postmigration factors such as acculturation stress from adapting to a new environment and socioeconomic challenges [28]. Many low- and middle-income countries, from which a substantial proportion of immigrants to Sweden originate, have undergone rapid epidemiological transitions characterized by increasing prevalence of obesity, diabetes, hypertension, tobacco use, and other CVD risk factors, driven by factors such as urbanization and lifestyle changes [11–13]. For example, diets high in saturated fat, salt, and sugar are widely reported in certain non-EU European, Asian, and Middle Eastern countries [14, 29, 30]. Consequently, some immigrants from these regions may arrive with an already elevated cardiovascular risk profile, especially those who have migrated in more recent decades. However, these premigration exposures are likely compounded by challenges encountered after migration. In addition to acculturative stress associated with adapting to a new social, cultural, and economic environment [28], immigrants may also experience structural barriers, including discrimination, structural racism, and unconscious bias within the society and healthcare. These experiences have been associated with chronic psychosocial stress and poorer cardiovascular health outcomes [31]. The gradual decline in IHD risk with increasing DoR may reflect improved socioeconomic and lifestyle integrations, greater familiarity with the healthcare system, and better access to preventive cardiovascular care and timely treatments [15, 32, 33]. Similarly, subgroup analyses by immigration cohort (i.e. by calendar period of immigration) showed that more recent male immigrants from the Middle East had the highest IHD risk, with risk increasing across successive immigration cohorts. This finding may reflect changing migration patterns, including a greater proportion of refugees and asylum seekers exposed to adverse social and health conditions before migration.

Overall, these findings suggest that the elevated IHD risk observed during the early years after migration is likely shaped by the interplay of cardiovascular risk accumulated before migration and social, economic, and healthcare-related experiences after resettlement. Future research should explore the mechanism underlying these inequalities, including the role of structural determinants and the broader social determinants of health that influence successful resettlement and long-term cardiovascular health.

Despite the cultural and socioeconomic similarities between Nordic nations and Sweden, immigrants from these countries exhibited a higher risk of IHDs than the host population. This suggests that lifestyle-related risk factors may play a more prominent role than differences in healthcare access or integration challenges, which may be more relevant for other immigrant groups. Persistent differences in lifestyle, including dietary habits, alcohol consumptions, physical activity, smoking, and other CVD risk factors such as hypertension and obesity, have been observed across the Nordic countries [34]. For example, historically higher levels of saturated fat intake, alcohol consumption, and smoking have been observed among Finns than Swedes, although these differences have narrowed in recent decades [35]. Similarly, Östergren *et al*. reported that Finnish migrants in Sweden exhibited intermediate lifestyle-related and CVD mortality patterns, with risks higher than those of Swedish-born individuals but lower than those of Finns living in Finland [36]. This pattern may reflect a gradual adoption of the host country’s health behaviors while indicating that premigration exposures and behavioral factors continue to influence cardiovascular health outcomes. We also observed a slightly elevated IHD among second-generation individuals of Nordic parental origin compared with native Swedes, suggesting that some of these risk patterns may persist across generations. Further research should explore specific characteristics, such as behavioral, environmental, and intergenerational factors that contribute to the elevated risk of IHD among Nordic immigrants and their descendants.

Changes in the composition of immigration to Sweden over recent decades may also partly explain the higher incidence rate of IHDs among some immigrant groups, particularly those with a short DoR. Although Sweden has continued to receive labor migrants, recent decades have also seen an increase in the number of refugees and asylum seekers from regions affected by war, conflict, and political instability. Compared with labor migrants, who are often younger and healthier because migration is primarily motivated by employment, refugees and asylum seekers represent a more heterogeneous population with respect to age and health status and are more likely to have experienced premigration stress and trauma and prolonged psychosocial stress, all of which may increase cardiovascular risk [33, 37]. In 2015 alone, for example, approximately 160 000 asylum seekers arrived in Sweden, mainly from conflict-affected regions in the Middle East and Asia [38]. Studies have shown that exposure to armed conflict is associated with an increased risk of stress-related mental health disorders [39], which are themselves associated with an increased risk of CVD [40]. Therefore, the elevated incidence of IHD among some recently arrived immigrant groups may partly reflect both premigration exposures and postmigration acculturation stress, which can persist for extended periods in some individuals.

We also observed that the ASIRs of IHDs declined over the past three decades, consistent with previous studies [3]. This decline is likely attributed to advancements in cardiovascular prevention and healthcare, including improved detection and management of major CVD risk factors such as hypertension and high cholesterol. However, the rate of decline varied across immigrant groups. ASIRs remained consistently higher among individuals from the Middle East and non-EU European countries than among Swedish-born individuals over the study period, highlighting the chronic and systemic nature of inequalities in cardiovascular health. Particularly among some immigrant groups, such as Asian men, ASIRs of IHD increased from being lower rates than those of Swedish-born individuals in the early 1990s to higher rates in recent years, suggesting an increasing cumulative burden of CVD risk factors over time within this immigrant populations.

### Strength and limitations

The high-quality Swedish national registers likely captured nearly all documented first-time IHD events during the study period, providing valid estimates of IHD incidence. We also presented country-specific estimates, as exposure to IHD risk factors may vary across immigrants’ countries and regions of origin. Moreover, the use of nationally representative data spanning more than three decades (1993–2023) enabled us to examine changes in IHD risk across successive immigration cohorts.

However, several limitations should be considered. First, the National Inpatient Register began in 1987, and complete data were available from 1990 onward to identify first-time IHD events. As a result, we cannot completely exclude individuals who experienced an IHD event before that date. Similarly, some immigrants may have experienced an IHD event prior to migration, which would not be captured in the Swedish registers. However, the 5-year buffer period we applied before follow-up commencement may reduce the likelihood of including individuals with a previous history of IHD. Second, the registers did not include important individual-level factors, such as lifestyle (e.g. smoking, alcohol consumption, and physical activity) and clinical risk factors (e.g. hypertension and diabetes), which could further modify the differences in IHD incidence across immigrant groups. Although our analyses were adjusted for socioeconomic factors—key determinants of CVD health and potential proxy for certain lifestyle factors—residuals confounding cannot be excluded. We also recommend further research to explore the role of these factors and to better understand the mechanisms underlying the observed differences in IHD risk. Third, ethnicity is not recorded in Swedish national registers. Therefore, immigrant background was defined using country of birth and parental country of birth, which serve as proxies for migration background but do not capture self-identified ethnicity. Consequently, our findings should be interpreted as reflecting migration- and origin-related characteristics rather than self-identified ethnicity.

## Conclusions

First-generation immigrants in Sweden experienced a higher risk of IHD than the host population, although the magnitude of this risk varied by country of origin, DoR, and immigration cohort. The elevated risks observed among recent immigrants and those with shorter DoR challenge the classical convergence hypothesis and suggest that the “healthy-migrant effect” may not apply uniformly across immigrant populations. These findings highlight the need for tailored cardiovascular prevention strategies and further research to better understand the mechanisms underlying immigrant inequalities in IHD.

## Supplementary Material

ckag192_Supplementary_Data

## Data Availability

The data underlying this article cannot be shared publicly due to the sensitive nature of individual-level health data. However, the data will be shared on reasonable request and with permission from the responsible authority.
