# Supplementary material for: First-time incidence of ischemic heart disease among immigrants in Sweden: variation by country of origin, duration of residence, and immigrant generation in a nationwide cohort study
Source: Eur J Public Health. 2026 Nov 6;36(6):ckag192. doi: 10.1093/eurpub/ckag192 (PMC13743345; doi:10.1093/eurpub/ckag192)
Supplement: Supplementary file 1 [file ckag192_supplementary_data.zip › ejph-2025-07-om-0564-File007.docx]

**Supplementary material**

**Table S1**. List of study variables and their analytical categories

| **Variable** | **Original register categories** | **Analytical categories** |
| --- | --- | --- |
| **Outcome** | | |
| Ischemic health disease (IHD) | ICD-9: 410–414; ICD-10: I20–I25 | First-time incident IHD |
|  |  |  |
| **Primary exposure variables** | | |
| Immigrant generation | Country of birth of the individual and parents | **Native Swedes**; born in Sweden to two Swedish-born parents  **Swedish-born individuals**; born in Sweden, regardless of parental country of birth  **First-generation immigrants (G1)** - born outside Sweden.  **Second-generation immigrants (G2)**; born in Sweden with at least one foreign-born parent |
| Country/region of origin | Country of birth (for G1) or parental country of birth (G2) | Nordic countries, non-EU Europe, EU (excluding Nordic countries), Middle East, Asia, Africa, North America, and Latin America; country-specific analyses where applicable |
| Duration of residence | Continuous years; calculated as the interval between the date of arrival and the end of follow-up | 5–15, 16–30, ≥31 years |
| Immigration cohort | Calendar year of arrival | Before 1970, 1971–2000, 2001–2010, 2011–2018 |
| **Adjustment covariates** | | |
| Age | Date of birth - calculated as the interval between the date of birth and cohort-entry | Used as the underlying time-scale in the Cox-regression models |
| Sex | Men; Women | Men; Women |
| Education | International Standard Classification of Education (ISCED) levels | Primary (ISCED 0-2), Secondary ((ISCED 3-4), Tertiary (ISCED 5-8), and missing/unknown |
| Income | Individual disposable income (Continuous) | Quintiles (Q1-Q5) |
| Marital status | Single, Married, Divorced, Widowed | Not married, Married, Divorced/Widowed |
| Occupational class | Three-digit International Standard Classification of Occupations (ISCO-08) code | **High-skilled white-collar**: Managers (major group 1), Professionals (2), Technicians and Associate Professionals (3)  **Low-skilled white collar:** Clerical Support Workers (4), Service and Sales Workers (5)  **High-skilled blue-collar**: Skilled Agricultural, Forestry and Fishery Workers (6), Craft and Related Trades Workers (7), Plant and Machine Operators and Assemblers (8)  **Low-skilled blue-collar:** Elementary Occupations (9) |
| Healthcare regions | Four-digit municipality code of residence. The first two digits identify the county and were used to define healthcare regions. | Stockholm-Götland, Uppsala-Örebro, Southeastern, Southern, Western, and Northern regions |

**Table S2.** Percentage and number of cases across the characteristics of the study population, stratified by sex and immigrant generation groups in Sweden, 1990 -mid-2023

| **Characteristics** | **Women: % (number of IHD cases)** | | | | **Men: % (number of IHD cases)** | | | |
| --- | --- | --- | --- | --- | --- | --- | --- | --- |
|  | All Sweden born (n=3,499,172) | Natives * (n=2,176,549) | G1  (n=784,519) | G2 * (n=396,532) | All Sweden born  (n=3,485,750) | Natives * (n=2,296,053) | G1  (n=767,276) | G2 * (n=417,567) |
| Age group (at cohort entry) |  | | | |  | | | |
| 35-50 | 62.6 (51691) | 86.1 (43380) | 77.4 (10616) | 77.2 (7649) | 66.7 (139957) | 86.5 (118604) | 80.8 (28830) | 78.3 (19784) |
| 51-65 | 16.9 (73825) | 13.9 (33649) | 14.4 (13966) | 22.8 (11383) | 16.9 (140518) | 13.5 (69954) | 13.9 (20790) | 21.7 (21853) |
| 66-85 | 20.5 (91168) | NA | 8.1 (8737) | NA | 16.4 (115299) | NA | 5.3 (7815) | NA |
| Mean age (SD) | 48.5 (15.5) | 40.1 (7.2) | 43.1 (11.7) | 38.9 (4.6) | 46.9 (14.5) | 40.0 (7.2) | 41.9 (10.3) | 38.8 (4.5) |
| Disposable income quintile (Q) |  | | | |  | | | |
| Q1 (lowest) | 19.7 (86507) | 5.1 (9701) | 21.4 (11597) | 7.8 (3013) | 19.2 (121710) | 6.3 (19554) | 23.7 (17854) | 10.9 (6343) |
| Q2 | 18.9 (614691) | 17.0 (26441) | 24.7 (11995) | 20.7 (7218) | 18.3 (103057) | 17.0 (52677) | 27.9 (19546) | 20.7 (13073) |
| Q3 | 19.2 (24588) | 24.3 (15128) | 23.7 (4082) | 22.4 (3177) | 19.4 (56805) | 23.5 (38308) | 22.7 (8698) | 21.5 (7422) |
| Q4 | 20.5 (17458) | 26.7 (10765) | 17.9 (2448) | 24.1 (2251) | 20.6 (49069) | 25.9 (33890) | 15.6 (5825) | 22.7 (6379) |
| Q5 (highest) | 21.7 (26662) | 26.9 (14994) | 12.3 (3197) | 25.4 (3373) | 22.4 (65133) | 26.8 (44129) | 10.1 (5512) | 24.0 (8422) |
| Education status |  | | | |  | | | |
| Primary completed | 27.4 (113856) | 14.8 (25595) | 24.4 (15733) | 18.9 (7260) | 28.2 (179481) | 19.8 (65507) | 21.9 (22232) | 23.2 (15746) |
| Secondary completed | 37.3 (71212) | 42.7 (35359) | 29.8 (10931) | 42.6 (8259) | 42.4 (146509) | 47.2 (82759) | 31.9 (22709) | 45.9 (17645) |
| Tertiary completed | 32.5 (26605) | 42.2 (15960) | 35.4 (4121) | 37.9 (3470) | 27.4 (64559) | 32.6 (39849) | 32.8 (11166) | 30.2 (8069) |
| Unknown/missing | 2.8 (5011) | 0.3 (115) | 10.4 (2534) | 0.5 (43) | 1.98 (5225) | 0.4 (443) | 13.3 (2328) | 0.7 (179) |
| Marital status |  | | | |  | | | |
| Single | 21.1 (18048) | 25.9 (9391) | 14.1 (1877) | 26.0 (2114) | 28.0 (56030) | 32.7 (34289) | 21.2 (5906) | 33.9 (7367) |
| Married | 42.1 (95076) | 49.2 (40941) | 49.2 (14729) | 44.0 (9536) | 51.5 (250075) | 50.4 (114694) | 54.7 (36002) | 46.8 (24699) |
| Divorced | 14.9 (33432) | 16.4 (16421) | 22.9 (8014) | 18.8 (4316) | 13.1 (55564) | 13.9 (32573) | 18.6 (12477) | 15.4 (7744) |
| Widowed | 21.2 (68797) | 8.4 (10264) | 11.7(8345) | 10.9 (3062) | 6.8 (32634) | 2.9 (6946) | 2.6 (2680) | 3.5 (1807) |
| Missing | 0.6 (1331) | 0.1 (12) | 2.2 (354) | 0.2 (4) | 0.7 (1471) | 0.1 (56) | 2.8 (370) | 0.3 (22) |
| Occupation class |  | | | |  | | | |
| White-collar: High skilled | 38.7 (30602) | 51.9 (21670) | 24.9 (3640) | 46.2 (4438) | 41.0 (101998) | 50.9 (72065) | 25.2 (10360) | 46.3 (13803) |
| White-collar: Low-skilled | 24.9 (46245) | 30.7 (28994) | 27.4 (6283) | 31.4 (6854) | 6.8 (21250) | 8.0 (13956) | 13.9 (5113) | 9.3 (3130) |
| Blue-collar: High skilled | 2.5 (4330) | 3.2 (2629) | 2.0 (493) | 2.9 (599) | 17.5 (61550) | 21.1 (40531) | 15.0 (7617) | 18.4 (7987) |
| Blue-collar: Low-skilled | 31.6 (129087) | 13.3 (23147) | 43.9 (22406) | 18.4 (6958) | 31.2 (197114) | 18.4 (59055) | 42.9 (33181) | 23.8 (15884) |
| Missing | 2.2 (6420) | 0.9 (589) | 1.7 (497) | 1.2 (183) | 3.4 (13862) | 1.6 (2951) | 2.9 (1164) | 2.1 (835) |
| Regions |  | | | |  | | | |
| Stockholm-Gotland | 23.8 (42049) | 23.7 (14265) | 36.5 (10523) | 30.4 (3980) | 23.4 (76843) | 23.4 (36094) | 36.4 (19076) | 30.3 (9337) |
| Uppsala-Örebro | 29.9 (66901) | 30.1 (24846) | 28.5 (9959) | 28.6 (5719) | 29.7 (121172) | 29.9 (59345) | 28.8 (17884) | 28.4 (12300) |
| Southeastern | 5.6 (13348) | 5.7 (4821) | 3.3 (930) | 4.3 (1137) | 5.6 (24567) | 5.7 (11822) | 3.3 (1790) | 4.2 (2326) |
| Southern | 19.8 (39664) | 20.1 (13919) | 18.4 (6538) | 19.5 (3407) | 19.8 (75360) | 20.0 (35057) | 18.6 (11543) | 19.4 (7681) |
| Western | 6.4 (15723) | 6.3 (5290) | 4.3 (1789) | 5.8 (1313) | 6.5 (28061) | 6.4 (12659) | 4.2 (2834) | 5.9 (2871) |
| Northern | 13.8 (37668) | 14.0 (13876) | 6.7 (3226) | 11.3 (3472) | 14.3 (68300) | 14.4 (33525) | 5.9 (3938) | 11.5 (7102) |
| Missing | 0.6 (1331) | 0.1 (12) | 2.2 (354) | 0.2 (4) | 0.7 (1471) | 0.1 (56) | 2.8 (370) | 0.3 (22) |

Abbreviations: G1 – First-generation immigrants, G2 – Second-generation individuals.

White-collar: High skilled jobs (Managers, Professions, and Technicians); White-collar: Low-skilled (Craft and Related Trades Workers and Skilled Agricultural, Forestry, and Fishery); Blue-collar: High skilled (Clerical Support Workers and Service and Sales Workers), and Blue-collar: Low-skilled (Plant and machine operators, and assemblers and Elementary occupations).

* The multi-generation register used to identify Natives and G2 individuals. This register links individuals born from 1932 onwards to their parents, provided both child and parent were registered in Sweden in 1961. For this reason, their maximum age at cohort in 1993 was 61

**Table S3:** Comparison of partially and fully adjusted hazard ratios (HRs) for first-time ischemic heart disease (IHD) by immigrant generation, country/region of origin, and sex, Sweden, 1993-mid-2023

| **Countries or regions** | **Women** | | **Men** | |
| --- | --- | --- | --- | --- |
|  | **Model 1** | **Model 2** | **Model 1** | **Model 2** |
| **Swedish-born** | Ref | Ref | Ref | Ref |
| **All G1** | 1.29 (1.27, 1.31) | 1.16 (1.14, 1.17) | 1.26 (1.25, 1.27) | 1.14 (1.13, 1.15) |
| **Nordic excl. Sweden** | 1.42 (1.39, 1.44) | 1.29 (1.27, 1.31) | 1.27 (1.26, 1.29) | 1.18 (1.17, 1.20) |
| Finland | 1.51 (1.48, 1.54) | 1.36 (1.33, 1.38) | 1.37 (1.35, 1.40) | 1.25 (1.23, 1.27) |
| Denmark | 1.13 (1.08, 1.18) | 1.09 (1.04, 1.14) | 1.06 (1.03, 1.10) | 1.02 (0.98, 1.05) |
| Norway | 1.25 (1.20, 1.30) | 1.14 (1.09, 1.18) | 1.14 (1.11, 1.19) | 1.13 (1.09, 1.17) |
| **EU & UK excl. Nordic** | 1.08 (1.05, 1.10) | 1.03 (0.99, 1.07) | 1.07 (1.06, 1.09) | 1.03 (1.01, 1.05) |
| Poland | 1.37 (1.31, 1.43) | 1.25 (1.19, 1.31) | 1.43 (1.37, 1.48) | 1.33 (1.28, 1.39) |
| Hungary | 1.26 (1.16, 1.35) | 1.17 (1.09, 1.27) | 1.17 (1.11, 1.23) | 1.09 (1.03, 1.14) |
| Romania | 1.04 (0.93, 1.17) | 0.93 (0.83, 1.04) | 1.11 (1.03, 1.21) | 0.98 (0.91, 1.05) |
| **Other European (non EU)** | 1.33 (1.29, 1.38) | 1.10 (1.06, 1.14) | 1.32 (1.29, 1.36) | 1.14 (1.11, 1.17) |
| Russia | 0.83 (0.68, 1.02) | 0.76 (0.63, 0.94) | 1.18 (0.97, 1.44) | 1.13 (0.93, 1.37) |
| Former Jugoslavia | 1.46 (1.40, 1.53) | 1.10 (1.06, 1.15) | 1.36 (1.32, 1.40) | 1.12 (1.08, 1.16) |
| Former Czechoslavkia | 1.01 (0.91, 1.13) | 1.06 (0.95, 1.18) | 1.11 (1.03, 1.20) | 1.13 (1.04, 1.21) |
| Former Soviet union | 1.36 (1.24, 1.48) | 1.31 (1.20, 1.43) | 1.21 (1.11, 1.32) | 1.18 (1.08, 1.28) |
| **Middle East** | 1.61 (1.56, 1.67) | 1.18 (1.14, 1.22) | 1.81 (1.77, 1.84) | 1.47 (1.44, 1.50) |
| Turkey | 1.63 (1.52, 1.74) | 1.21 (1.13, 1.29) | 1.76 (1.69, 1.84) | 1.44 (1.38, 1.50) |
| Iraq | 1.73 (1.62, 1.86) | 1.17 (1.09, 1.26) | 1.88 (1.81, 1.95) | 1.46 (1.41, 1.51) |
| Iran | 1.41 (1.32, 1.50) | 1.16 (1.09, 1.25) | 1.54 (1.48, 1.60) | 1.37 (1.32, 1.43) |
| Lebanone | 1.72 (1.53, 1.94) | 1.17 (1.04, 1.32) | 2.04 (1.92, 2.17) | 1.55 (1.46, 1.65) |
| Syria | 1.81 (1.64, 1.99) | 1.23 (1.12, 1.36) | 2.29 (2.17, 2.41) | 1.74 (1.65, 1.83) |
| **Africa** | 0.74 (0.67, 0.82) | 0.61 (0.55, 0.68) | 0.91 (0.87, 0.96) | 0.79 (0.75, 0.83) |
| **Asia** | 0.99 (0.93, 1.06) | 0.78 (0.73, 0.83) | 1.39 (1.34, 1.46) | 1.17 (1.12, 1.22) |
| Afganistan | 2.12 (1.76, 2.57) | 1.38 (1.15, 1.67) | 1.84 (1.62, 2.09) | 1.42 (1.25, 1.61) |
| India | 1.55 (1.31, 1.84) | 1.27 (1.07, 1.51) | 1.77 (1.61, 1.95) | 1.48 (1.35, 1.62) |
| Pakistan | 2.96 (2.39, 3.67) | 1.79 (1.45, 2.22) | 2.71 (2.44, 3.01) | 1.93 (1.74, 2.14) |
| Sri Lanka | 1.83 (1.47, 2.29) | 1.26 (0.99, 1.56) | 2.09 (1.77, 2.48) | 1.63 (1.37, 1.91) |
| **North America** | 0.82 (0.69, 0.98) | 0.78 (0.66, 0.94) | 0.87 (0.77, 0.97) | 0.83 (0.74, 0.93) |
| **Latin America** | 0.78 (0.72, 0.84) | 0.72 (0.66, 0.77) | 0.74 (0.70, 0.78) | 0.69 (0.65, 0.73) |
|  | | | | |
| **Natives** | Ref | Ref | Ref | Ref |
| **All G2** | 1.14 (1.12, 1.17) | 1.11 (1.09, 1.14) | 1.12 (1.10, 1.14) | 1.08 (1.06, 1.09) |
| Nordic | 1.19 (1.13, 1.25) | 1.14 (1.09, 1.20) | 1.11 (1.07, 1.14) | 1.07 (1.04, 1.11) |
| EU and UK | 0.95 (0.87, 1.04) | 1.05 (0.96, 1.15) | 0.98 (0.92, 1.03) | 1.03 (0.97, 1.08) |
| Other Europe | 1.04 (0.86, 1.27) | 1.10 (0.91, 1.34) | 1.08 (0.96, 1.21) | 1.12 (0.99, 1.26) |
| Middle East | 1.68 (0.90, 3.13) | 1.58 (0.85, 2.94) | 1.69 (1.20, 2.37) | 1.31 (0.89, 1.93) |

Model 1 adjusted for calendar period (as time-varying covariate); Model 2 adjusted for model + socioeconomic factors (disposable income, educational attainment, marital status, occupation class, and health care regions).

G1 - First generation immigrants. G2 - Second-generation individuals.

**Table S4:** Comparison of partially and fully adjusted hazard ratios (HRs) for first-time ischemic heart disease (IHD) by duration of residence, region of origin, and sex among first-generation immigrants aged 35-85 compared with Swedish-born individuals, 1993-mid-2023

| **Regions** | **DoR (in years)** | **Women** | | **Men** | |
| --- | --- | --- | --- | --- | --- |
|  |  | **Model 1** | **Model 2** | **Model 1** | **Model 2** |
| **All G1** | 5 to 15 | 3.86 (3.73, 4.00) | 3.67 (3.53, 3.82) | 4.24 (4.14, 4.34) | 4.13 (4.03, 4.25) |
|  | 16 to 30 | 2.02 (1.97, 2.07) | 1.79 (1.74, 1.84) | 2.51 (2.46, 2.55) | 2.28 (2.24, 2.32) |
|  | 31+ | 1.05 (1.03, 1.06) | 0.98 (0.96, 0.99) | 0.90 (0.89, 0.91) | 0.86 (0.85, 0.87) |
| **Nordic excl. Sweden** | 5 to 15 | 3.96 (3.58, 4.37) | 3.46 (3.13, 3.83) | 3.02 (2.82, 3.23) | 3.04 (2.83, 3.26) |
|  | 16 to 30 | 4.63 (4.43, 4.84) | 3.97 (3.79, 4.15) | 3.50 (3.39, 3.62) | 3.26 (3.15, 3.37) |
|  | 31+ | 1.26 (1.24, 1.28) | 1.15 (1.13, 1.17) | 1.07 (1.06, 1.09) | 1.01 (0.99, 1.02) |
| **EU & UK excl. Nordic** | 5 to 15 | 3.07 (2.82, 3.32) | 2.54 (2.34, 2.76) | 2.94 (2.78, 3.11) | 2.57 (2.42, 2.73) |
|  | 16 to 30 | 1.83 (1.73, 1.94) | 1.55 (1.47, 1.64) | 2.12 (2.03, 2.21) | 1.93 (1.86, 2.02) |
|  | 31+ | 0.87 (0.85, 0.91) | 0.86 (0.83, 0.88) | 0.85 (0.83, 0.87) | 0.83 (0.81, 0.85) |
| **Other European (non EU)** | 5 to 15 | 4.61 (4.21, 5.05) | 3.69 (3.36, 4.06) | 6.66 (6.23, 7.11) | 5.37 (5.02, 5.74) |
|  | 16 to 30 | 1.71 (1.59, 1.82) | 1.33 (1.24, 1.43) | 2.15 (2.05, 2.25) | 1.72 (1.65, 1.81) |
|  | 31+ | 0.99 (0.94, 1.04) | 0.84 (0.80, 0.88) | 0.92 (0.89, 0.95) | 0.81 (0.78, 0.84) |
| **Middle East** | 5 to 15 | 5.27 (4.99, 5.56) | 3.73 (3.52, 3.95) | 6.19 (5.97, 6.04) | 4.79 (4.26, 4.97) |
|  | 16 to 30 | 1.78 (1.69, 1.88) | 1.24 (1.18, 1.31) | 2,76 (2.68, 2.84) | 2.08 (2.02, 2.14) |
|  | 31+ | 0.57 (0.52, 0.62) | 0.47 (0.43, 0.51) | 0.70 (0.67, 0.73) | 0.62 (0.60, 0.65) |
| **Africa** | 5 to 15 | 1.56 (1.31, 1.87) | 1.13 (0.95, 1.36) | 2.05 (1.85, 2.26) | 1.50 (1.36, 1.66) |
|  | 16 to 30 | 0.84 (0.71, 0.99) | 0.67 (0.56, 0.79) | 1.76 (1.63, 1.89) | 1.41 (1.30, 1.52) |
|  | 31+ | 0.37 (0.29, 0.46) | 0.32 (0.26, 0.40) | 0.51 (0.47, 0.55) | 0.46 (0.42, 0.49) |
| **Asia** | 5 to 15 | 2.61 (2.31, 2.95) | 1.81 (1.59, 2.04) | 3.91 (3.60, 4.25) | 3.00 (2.76, 3.27) |
|  | 16 to 30 | 1.16 (1.04, 1.28) | 0.81 (0.73, 0.89) | 2.17 (2.03, 2.31) | 1.71 (1.61, 1.84) |
|  | 31+ | 0.56 (0.50, 0.64) | 0.47 (0.42, 0.54) | 0.76 (0.70, 0.81) | 0.65 (0.60, 0.69) |
| **North America** | 5 to 15 | 1.92 (1.19, 3.09) | 1.47 (0.92, 2.37) | 2.67 (2.04, 3.48) | 2.28 (1.75, 2.98) |
|  | 16 to 30 | 1.06 (0.75, 1.47) | 0.88 (0.63, 1.23) | 1.24 (1.02, 1.50) | 1.12 (0.92, 1.36) |
|  | 31+ | 0.52 (0.38, 0.70) | 0.52 (0.38, 0.69) | 0.52 (0.43, 0.63) | 0.49 (0.41, 0.60) |
| **Latin America** | 5 to 15 | 3.27 (2.79, 3.84) | 2.76 (2.35, 3.24) | 2.93 (2.53, 3.39) | 2.59 (2.24, 3.01) |
|  | 16 to 30 | 1.29 (1.15, 1.45) | 1.12 (0.99, 1.26) | 1.80 (1.66, 1.96) | 1.50 (1.38, 1.64) |
|  | 31+ | 0.33 (0.28, 0.38) | 0.29 (0.25, 0.34) | 0.38 (0.35, 0.42) | 0.36 (0.33, 0.40) |

**Model 1** adjusted for calendar period (as time-varying covariate); **Model 2** adjusted for model + socioeconomic factors (disposable income, educational attainment, marital status, occupation class, and healthcare regions).

G1 – First-generation immigrants; DoR – Duration of residence
